# ‘Mother(Nature) knows best’ – hijacking nature-designed transcriptional programs for enhancing stress resistance and protein production in *Yarrowia lipolytica*; presentation of YaliFunTome database

**DOI:** 10.1186/s12934-023-02285-x

**Published:** 2024-01-18

**Authors:** Maria Gorczyca, Wojciech Białas, Jean-Marc Nicaud, Ewelina Celińska

**Affiliations:** 1 Department of Biotechnology and Food Microbiology, Poznan University of Life Sciences, 60-637 Poznań, Poland; 2 Université Paris-Saclay, INRAE, AgroParisTech, Micalis Institute, 78350 Jouy-en-Josas, France

**Keywords:** Yeast, Transcription factors, Stress resistance, Protein production, Global metabolic engineering, *Yarrowia* cultivation protocol

## Abstract

**Background:**

In the era of rationally designed synthetic biology, heterologous metabolites production, and other counter-nature engineering of cellular metabolism, we took a step back and recalled that ‘Mother(-Nature) knows best’. While still aiming at synthetic, non-natural outcomes of generating an ‘over-production phenotype’ we dug into the pre-designed transcriptional programs evolved in our host organism—*Yarrowia lipolytica*, hoping that some of these fine-tuned orchestrated programs could be hijacked and used. Having an interest in the practical outcomes of the research, we targeted industrially-relevant functionalities—stress resistance and enhanced synthesis of proteins, and gauged them over extensive experimental design’s completion.

**Results:**

Technically, the problem was addressed by screening a broad library of over 120 *Y. lipolytica* strains under 72 combinations of variables through a carefully pre-optimized high-throughput cultivation protocol, which enabled actual phenotype development. The abundance of the transcription program elicitors—transcription factors (TFs), was secured by their overexpression, while challenging the strains with the multitude of conditions was inflicted to impact their activation stratus. The data were subjected to mathematical modeling to increase their informativeness.

The amount of the gathered data prompted us to present them in the form of a searchable catalog – the YaliFunTome database (https://sparrow.up.poznan.pl/tsdatabase/)—to facilitate the withdrawal of biological sense from numerical data. We succeeded in the identification of TFs that act as omni-boosters of protein synthesis, enhance resistance to limited oxygen availability, and improve protein synthesis capacity under inorganic nitrogen provision.

**Conclusions:**

All potential users are invited to browse YaliFunTome in the search for homologous TFs and the TF-driven phenotypes of interest.

**Supplementary Information:**

The online version contains supplementary material available at 10.1186/s12934-023-02285-x.

## Background

Any rationally designed biotechnological production process artificially forces the producer cell to the non-natural ‘over-production phenotype’ state. It is now well-recognized that such a phenotype is elicited by a concerted action of multiple genes/molecular identities. Hence, designing such a phenotype operating under specific process conditions requires a thorough understanding of the molecular and regulatory mechanisms governing the target bioproduct synthesis; and then—using this knowledge to orchestrate genotype and environment towards the objective functionality. With the current technology progress, reaching the “proof-of-concept” level of microbial cell factory readiness can be relatively rapidly accomplished. However, its fine-tuning to reach actual industrial relevance requires detailed insight into the host’s system. Such a goal can be achieved by either a very extensive metabolic engineering strategy (which will inevitably impose a high metabolic burden on the engineered cell) or by hijacking nature-pre-designed programs governed by native global regulators.

The concept of using global regulators for engineering complex traits in yeast (like stress resistance or over-production of proteins) has been already proposed in several different forms. Alper and Stephanopoulos [1] proposed ‘global transcription machinery engineering’ for optimizing complex target phenotypes, and implemented the idea by conducting the sigma70 subunit of RNA polymerase-based engineering in *Saccharomyces cerevisiae*. Overexpression of the Unfolded Protein Response (UPR)-activating Transcription Factor (TF), Hac1, was the most frequently employed strategy for enhancing recombinant proteins (r-Prots) production. Hac1’s direct implication in the regulation of endoplasmic reticulum (ER)-resident events and restoring ER homeostasis made it the most straightforward target in such endeavors. It was demonstrated that overexpression of *HAC1* improves the secretion of r-Prots in *S. cerevisiae* [2], *Pichia pastoris* (renamed to *Komagataella phaffi*) [3, 4], and recently in *Yarrowia lipolytica* [5, 6]. A complementary approach targeted Hsf1 (heat shock factor 1), which is the key regulator of heat shock response and governs the “cytosolic” version of UPR [7, 8]. Hou et al. [9] investigated the regulome of *S. cerevisiae*’s Hsf1 and showed that it is mainly represented by molecular chaperones involved in protein folding, thus preventing the accumulation of misfolded or aggregated proteins. Continuous activation of heat shock response by overexpression of mutant *HSF1*-R206S triggered increased production of native and r-Prots [9]. Global regulators engineering was also employed to attenuate endogenous oxidative stress emerging from the oxidative folding process. Hap1 is a single TF managing aerobic metabolism in *S. cerevisiae*. It is responsible for sensing the oxygen levels via the heme signaling pathway, and activation of the oxidative stress response genes [10]. Overexpression (OE) of *HAP1* in *S. cerevisiae* overproducing a secretory r-Prot led to the mitigation of the negative effects caused by reactive oxygen species accumulation, hence increasing the production capacity of the strain [11]. Very recently, synthetic activation of the general stress response TF Msn4 (and its synthetic version synMsn4, alone or in combination) triggered over fourfold enhancement in r-Prot production [12].

Considering its industrially-relevant metabolic characteristics, its genetic amenability, and its genomic sequence available [13], *Y. lipolytica* has emerged as a robust biotechnological production platform of many added-value bioproducts [14]. Both native and heterologous molecules have been produced with this yeast platform, including organic acids [15], polyols [16], aromas [17, 18], carotenoids [19, 20], usual and unusual fatty acids [21, 22] etc. With the advent of numerous tools and the progress in understanding its molecular background, *Y. lipolytica* has also become a recognized platform for the production of r-Prots [23–25].

To date, the research on TFs in *Y. lipolytica* has been largely conducted in the context of basic studies. Most of the literature reports refer to an individual TF’s role in the regulation of dimorphic transition; implication in this phenomenon has been proved for Hoy1 [26], Mhy1 [27, 28], Bmh1 [29], YAP-like YALI0D07744g [30], Znc1 [31], and Msn2 [32]. The role in the regulation of lipid metabolism in *Y. lipolytica* has been documented for several other TFs, Yas1 and Yas2 [33], Yas3 [34], Por1 [35], Mig1 [36], and Gzf2 and Gzf3 [32]. The actual implementation of the ‘global transcription machinery engineering’ concept in this species has been to date limited to only several reported studies; for example, high-throughput functional screens of over one hundred twenty strains individually over-expressing (OE-ing) TFs that were conducted to fish out global regulators involved in lipids accumulation [37, 38]. Recently, [39] studied the impact of five selected TFs Hsf1, Gzf1, Crf1, Skn7, and YAP-like (YALI0D07744g) on two interconnected complex traits—stress resistance and r-Prots synthesis. OE of Yap-like TF was proven to alleviate growth retardation under high pH, while Gzf1 and Hsf1 were shown to serve as universal enhancers of r-Prot production in *Y. lipolytica*. On the other hand, deletion of *SKN7* and *HSF1* disabled growth under hyperosmotic stress.

As recently pointed out by the inventors of the Yeastract + database [40, 41], the lack of integration of regulatory information in the global metabolic models hinders their predictive ability. Hence, this functionality has been implemented in a recent update of the database [41]. However, due to under-investigation, the information load for *Y. lipolytica* in this context is rather scarce. Considering how huge potential is held by ‘global transcription machinery engineering’ we decided to explore this niche and provide experimental evidence on the actual implications of *Y. lipolytica*’s TFs in stress resistance and/or in the synthesis of r-Prots. Knowing that TF’s activity is not secured by the sole abundance of the protein, we challenged the strains constitutively OE-ing the TFs with a combination of environmental stress factors. Altogether, 125 *Y. lipolytica* strains individually OE-ing one of the TF-encoding genes were screened under 72 different conditions. We read growth and the level of a reporter r-Prot synthesis, which was used as a gauge for a global protein synthesis capacity—comprising, but not limited to r-Prots. The amount and informativeness of the data we gathered from completing our experimental design inspired us to present them in the form of a searchable catalog—the YaliFunTome database, to facilitate the withdrawal of biological sense from extensive sets of numerical data.

## Materials and methods

### Data collection and analysis

#### Biological material

A collection of 125 *Y. lipolytica* strains over-expressing (OE) individually one of the TFs identified in *Y. lipolytica*’s genome, and a reporter protein (RedStar2) was used in this study. The search of the TFs within the *Y. lipolytica* genome and construction of the strains co-over-expressing (co-OE) TF and a reporter r-Prot was done previously [37]. Both genes were cloned under a constitutive promoter pTEF, and were integrated in a zeta platform at *URA3* locus. The collection contained two control strains: (i) a completely prototrophic version of the parental strain (JMY2900); and (ii) complete prototroph OE the reporter r-Prot solely (JMY2810). The former was used as a control of burden imposed by OE of the reporter r-Prot and control of cross-contamination between cultures. The latter was considered the actual “control strain” used for normalization of the data from the TF-OE strains. All the strains were constructed on the background of the JMY2566 strain. More technical details on the cloning strategy can be found in [42].

All the strains were deposited as 15% glycerol stocks at − 80 °C for long-term storage. While running the experimental plan, the strain collection was freshly plated from stocks to YPD-agar medium (g L^−1^: yeast extract, 5 (BTL, Łódź, Poland); peptone, 10 (BTL); glucose, 20 (POCH, Gliwice, Poland); solidified with agar, 15 (BTL)) every 2 weeks.

### Experimental design

**Fig. 1:**
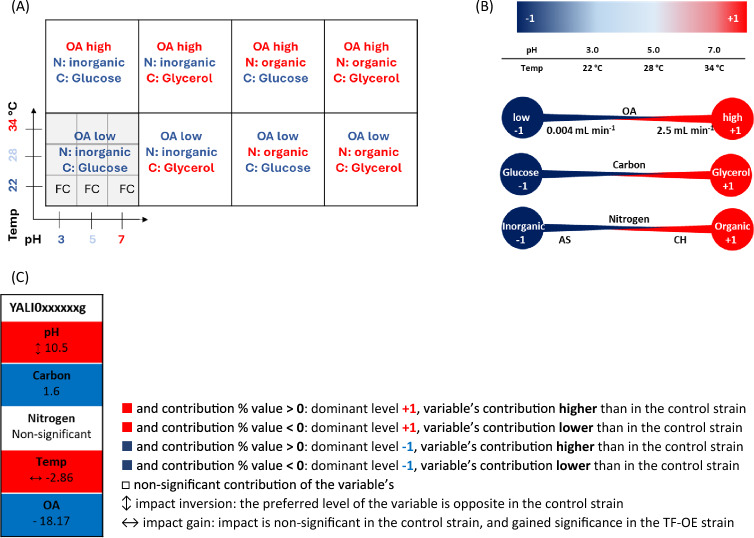
A system for graphical presentation of the data and variables coding. Graphical presentation of the arrangement of the variables’ combinations corresponding to the data presentation scheme (**A**), and the variables’ coding system (**B**), exemplary Factor’s Contribution ranking with a legend (**C**). The coding system corresponds to Table 1. The rankings are prepared based on Response Surface Models. Each ranking represents the variable’s contribution to a response of a given read parameter (growth/r-Prot/normalized r-Prot) elicited by a specific TF-OE strain. CH—casamino acid hydrolysate, AS—ammonium sulfate, OA- oxygen availability, FC–fold change of the response from the TF-OE strain over the control strain

The experimental plan was designed using the Design of Experiments (DoE) methodology in DesignExpert software (StatSoft, Tulsa, USA) as a Face-centered Central Composite Design. It implemented five external variables: two numerical (continuous) and three categoric (discrete), as presented in Table 1. Variables were technically implemented as follows: numerical variables: (i) pH (at levels: 3.0, 5.0, or 7.0) adjusted during the media preparation with 20% NaOH (w w^−1^) and by change of the buffer molarity (pH 3, − 0.1 M maleic acid buffer, pH 5 and 7, − 0.2 M maleic acid buffer); (ii) temperature (22, 28 and 34 °C) by setting thermostat; categoric variables; (iii) oxygen availability (OA) changed by use of different sandwich covers [43]: high OA (2.5 mL min^−1^ headspace air exchange), low OA (0.004 mL min^−1^); (iv) carbon source—glucose (encoded − 1) or glycerol (encoded + 1); and (v) nitrogen source: inorganic (ammonium sulfate; encoded − 1) or organic (casamino acid hydrolysate; encoded + 1). All the combinations resulted in 72 variants of environmental conditions, 8 of which were considered ‘central variants’ (according to the heatmaps: these were pH 5 and temp 28 °C with different categoric factors combinations—OA, carbon, and nitrogen). Each environmental variant was conducted in biological duplicate, but the 8 ‘central variants’ were conducted in pentaplicate, resulting in 64 ‘outer variants’ plus 8 ‘central variants’ × 5 = 104 culture runs. Each of the 104 culture runs was conducted in two biological repetitions. The order of culture execution was randomized. Figure 1 schematically illustrates the variables combinations, corresponding to the data presentation scheme.

**Table 1:** Summary of experimental setup designed with DoE (DX, StatSoft)

Design type: face-centered Central composite design					Runs: 104 for each of 125 TF = 13 000 in duplicate = 26,000 readouts on FL (fluorescence) and growth (OD600)					
The central variants were repeated five times. The order of culture execution was randomized										
Process factors		Categoric: A, B, C Numeric: d, e								

Factor	Name	Units	Change	Type	Minimum	Maximum	Coded Low	Coded High	Mean	Std. Dev
A	AIR “limiting”	0/1	Easy	Categoric	Low	High			Levels: 2	
B	Carbon source	Glu/Gly	Easy	Categoric	Glucose	Glycerol			Levels: 2	
C	Nitrogen source	i/o	Easy	Categoric	Inorganic: Ammonium Sulfate	Organic: Casamino Acid Hydrolysate			Levels: 2	
d	pH	pH	Hard	Numeric	3	7	− 1 → 3	+ 1 → 7	5	2.83
e	Temperature	°C	Hard	Numeric	22	34	− 1 → 22.00	+ 1 → 34.00	27.1	5.443

### High-throughput screens

#### Media preparation

The composition of the culture media was carefully adjusted to minimize background fluorescence (FL), provide sufficient amounts of carbon (C) and nitrogen (N) to avoid starvation, and maintain the pH stably throughout the culturing time. The medium was composed as follows [g L^−1^]: yeast nitrogen base without amino acids and ammonium sulfate, 5.1 (Sigma-Aldrich); ammonium sulfate or casamino acid hydrolysate, 15, (POCH); glucose or glycerol, 35 (POCH); buffered with 0.1 or 0.2 M maleic acid (POCH). Precultures were developed in media composed of a yeast nitrogen base, 5.1 (Sigma-Aldrich); ammonium sulfate, 15, (POCH); glucose, 25 (POCH); buffered with 0.2 M maleic acid at pH 5.0. All the media were filter-sterilized with 0.22-μm filters (Merck- Millipore, Darmstadt, Germany).

#### Culture conditions

Since typical multitier plates were found useless for reliable high-throughput screens and phenotype reads of *Y. lipolytica* strains, we used 24-well Duetz-System square plates in 2.5 mL working volume (EnzyScreen BV, Netherlands), agitated at 250 rpm. The cultures were continued for 48 h and samples were collected at the end of the cultivation time (the timing was pre-optimized). Precultures were developed for 18 h at 28 °C, under high OA. The main cultures were inoculated at 4% (v v^−1^).

#### Analytical methods

Samples were analyzed for growth and FL from the reporter protein (RedStar2) following dilution in 0.75% NaCl (POCH) to match a linear range of the methods. Absorbance was measured at 600 nm in transparent 96-well plates (Costar; Merck). FL was determined under at ex/em 550/595 nm in black opaque plates (Thermo Fisher Scientific) Both measurements were done using a Tecan Spark automatic plate reader (Tecan Group Ltd., Mannedorf, Switzerland).

### Data processing and statistical analyses

Results for total FL from the reporter r-Prot are expressed in Fluorescence Units (FU); growth is expressed in absorbance units from measuring optical density at 600 nm wavelength. Normalized r-Prot measure was calculated by dividing the FU value per OD600 value giving the specific fluorescence (RFU–relative fluorescence units). Statistical significance of the difference in a given measure between a TF-OE strain and the control strain was assessed by analysis of variance (ANOVA) test, with significance level set at p-value < 0.05 (Statistica, StatSoft-Tibco, Tulsa, USA). Fold change (FC) values express the ratio of a given measure for a TF-OE strain over the control strain.

Mathematical models describing the growth, total r-Prot synthesis, and normalized r-Prot synthesis by the strains within the range covered by the variables were developed using raw data (not FC) response surface methodology (RSM) in DesignExpert software. It resulted in a set of the following items for each of the TF-OE strains: (i) a model equation (including interactions between the variables; equations were not shown); (ii) set of eight 3D contour plots covering all the responses within the range of tested variables (arranged as in Fig. 1; color-coded according to the legend); and (iii) quantitative assessment of the variables contribution to the response (Factor’s Contribution Tables; percentage value). In the latter, after manual inspection of the data, the interactions between the variables were ignored (for clarity of data presentation). The significance of each variable was assessed by ANOVA tests, at p-value < 0.05. To compare the contribution of individual variables in the TF-OE strain model and the control, the values obtained for the former were normalized over the latter. The higher/lower contribution was indicated by a positive/negative value of the percentage contribution, while the direction of contribution is color-coded/labeled according to the legend. All of the obtained models were found significant, fitting of data was color-coded.

### YaliFunTome database construction

The YaliFunTome database was created using the MariaDB relational database management system (https://mariadb.org/), PHP 7.4.33 (https://www.php.net/), Bootstrap 5.3.1 (https://getbootstrap.com/), HTML 5, CSS 3, JavaScript and jQuery 3.7.0 (https://jquery.com/). The database has been tested on several different web browsers including Google Chrome, Mozilla Firefox and Microsoft Edge.

## Results and discussion

### Getting the pipeline working

Having a strictly aerobic, filamenting-upon-stress biological object in our hands, it rapidly appeared that the majority of high-throughput cultivation protocols working for the other yeast species are non-operable in this case. In addition, due to the modification of global regulators, the set of 125 strains analyzed here comprised those showing no obvious aberrant phenotype under the majority of conditions, and those severely affected in basic metabolism. Hence, it was necessary to develop a new high-throughput cultivation protocol that will enable reliable examination of all the phenotypes in a streamlined and normalized manner.

First, we defined a set of variables that are relevant to the *Y. lipolytica* r-Prots overproducer phenotype, and which could be affected by the TFs’ OE. The primary limitation encountered in *Y. lipolytica* cultures is limited oxygen provision, staying beyond the technical capabilities of laboratory or industrial equipment. Recently, [44] showed that Oxygen Availability (OA) is the key factor determining the rate of r-Prot synthesis. The same conclusions were made for the production of organic acids and polyols [45–47]. Similarly, it has been evidenced that pH is a strong modulator of the r-Prots-producing capacity in this species [48]. While the pH level can be relatively easily stabilized in bioreactor cultivations, local fluctuations in medium acidity are known to occur. Followingly, it was demonstrated that elevated temperature treatment or synthetic induction of heat shock response (by OE of *HSF1* TF) both had a positive impact on the r-Prots synthesis rate in *S. cerevisiae* [9]. In contrast, heat-shock treatment was found detrimental to r-Prots synthesis in *Y. lipolytica*, and only a low-temperature treatment exerted a positive impact in this regard [49]. Still, OE of *HSF1* in *Y. lipolytica* led to enhancement in r-Prot synthesis [6], correspondingly to what was found for *S. cerevisiae*. Therefore, we hypothesize that the OE of some TFs may mitigate the adverse effects of non-optimal OA, pH, and temperature.

In addition, previous investigations into *Y. lipolytica*’s biology clearly showed that the type of carbon source (glucose-glycerol) had a tremendous impact on the evoked regulatory mechanisms [37, 50]. Likewise, the type and concentration of available nitrogen sources were shown to have a fundamental impact on *Y. lipolytica* biology [51], including r-Prots production [52, 53] and dimorphic transition [54, 55].

**Fig. 2:**
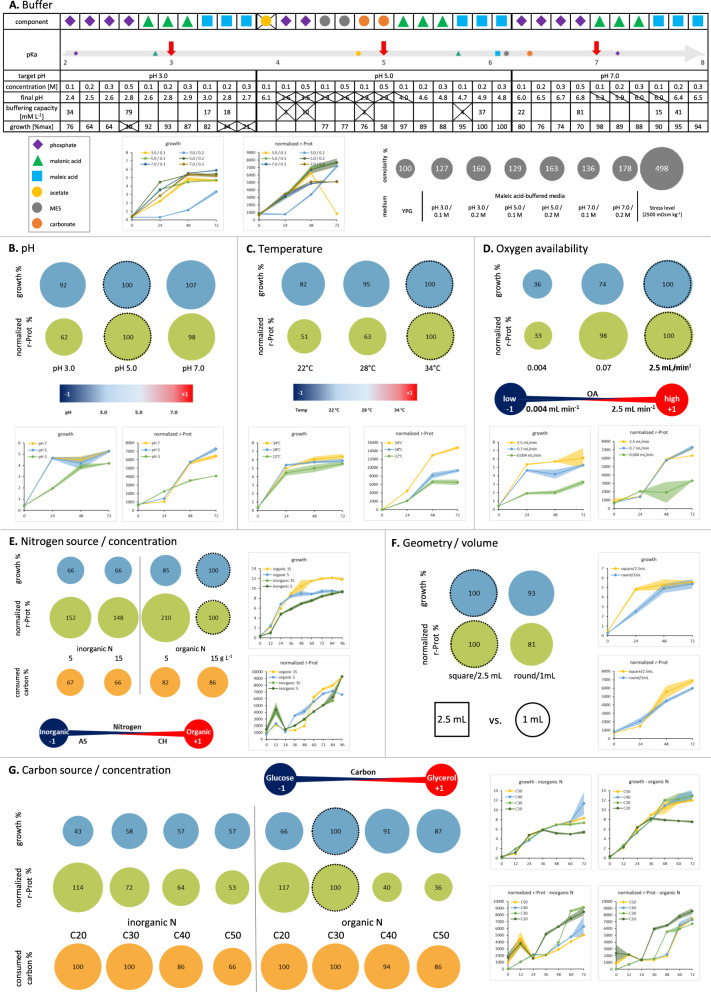
Main findings of the high-throughput cultivation protocol’s optimization. **A** Buffer selection—six different chemical compound combinations with buffering properties were tested. The top row in the table represents the composition of the buffer encoded by symbols (in the legend); the buffers’ pKa values are marked with these symbols on the grey arrow (target pH values marked with red arrows). Some components have two pKa values making them more suitable for covering the whole span of tested pHs (it is presumed that 1 value away from the pKa value the buffer is still in its range of buffering capacity). Initially set pH, molarity, and the experimentally defined post-culture pH (control strain, main culture medium, 72 h) are given as numbers. ‘Buffering capacity’ [M L^−1^], and percentage of maximal growth [%max] at 48h of cultivation (a proxy for growth impairment) are given as ‘bubble graphs’. Boxes crossed out indicate that the buffer did not meet requirements in terms of (i) component—utilization by *Y. lipolytica* (based on HPLC data), (ii) final pH—drop in pH > 1, (iii) buffering capacity—buffering capacity level < 10 M L^−1^, iv) growth—pronounced growth impairment < 40% of maximal growth. Kinetic graphs represent growth and normalized r-Prot for *Y. lipolytica* control strain grown in a maleic-acid buffered medium (3 pH values, 2 molarities). Grey ‘bubble plots’ represent osmolarity readouts of YPG medium buffered with the indicated maleic acid buffer, and the osmolarity level known to impose severe stress for *Y. lipolytica* cells. **B**, **C** Continuous variables range examination (pH and temperature)—these sections represent pH and temp as numerical, continuous parameters coded from -1 (pH 3.0 or 22 °C) to + 1 (pH 7.0 or 34 °C). Bubble plots represent growth and normalized r-Prot as % values of these parameters read under pH 5.0 or 34 °C for the control strain (48 h). Kinetic graphs represent growth and normalized r-Prot for *Y. lipolytica* control strain in time. **D**, **E**, **G** Discrete variables levels examination (Oxygen availability, nitrogen, and carbon source types and concentrations)—these sections represent OA, N, and C as categoric parameters encoded as -1 (low OA/inorganic N/glucose) or + 1 (high OA/organic N/glycerol) levels. Oxygen availability variation was implemented by the use of Duetz system sandwich covers of different headspace volume exchange rates: 0.004, 0.7, and 2.5 mL min^−1^. Optimization of Nitrogen source and concentration was conducted with inorganic N (AS–ammonium sulfate) or organic N (CH – casamino acid hydrolysate) substrates at concentrations of 5 or 15 g L^−1^. Optimization of carbon source concentrations was performed with glucose at concentrations: 20, 30, 40, and 50 g L^−1^, marked as C20, C30, C40, and C50, respectively—and cultivated on either one of two N sources. Consumed carbon in these cultures was calculated as residual C amount based on HPLC data from 48h of cultivation in relation to its initial load. Kinetic graphs represent growth and normalized r-Prot for *Y. lipolytica* control strain in time. **F** Geometry/volume—this section presents a comparison of two types of 24-well plates with different geometry of wells (square/round) and working volume recommended by the manufacturer (2.5/1 mL). Bubble and time-point plots are constructed as in other sections. Kinetic plots: x-axis time [h] and the y-axis OD_600_ (optical density at 600nm) value or sFL [FL/OD_600_] (specific fluorescence) for growth and normalized r-Prot, respectively. Shaded areas represent standard deviation (SD) values, based on biological duplicates or triplicates. All bubble plots are calculated in percentage to the highest or optimal variant (variants 100% are framed with black-dashed lines) in 48 h of cultivation. The percentage value is indicated within the bubble and corresponds to the bubble area. If not stated otherwise, cultures were performed in high OA, 28 °C, pH 5.0, and with glucose (20 g L^−1^) and ammonium sulfate (15 g L^−1^) as carbon and nitrogen sources in 24-well plates with JMY2810 as the control strain

The abovementioned conditions were then tested in an extensive set of preliminary experiments checking the feasibility and mode of the variables’ implementation. Optimization studies were conducted with two control strains—JMY2810 overexpressing a fluorescent reporter r-Prot, and a complete prototroph JMY2900, to estimate the burden imposed by the sole reporter r-Prot OE. The strains were analyzed for growth and, in the case of JMY2810, for protein synthesis (gauged by the reporter r-Prot fluorescence). As the burden imposed by the reporter r-Prot synthesis was not statistically significant in terms of growth, results for JMY2900 were not shown. The results of the preliminary optimization tests indicated that (Fig. 2):

i. Growth and r-Prots synthesis is supported within the pH range of 3 to 7, which can be effectively implemented and stably maintained at a desired level by using maleic buffer; this buffer type fulfills the following requirements: (i) spans the whole range to be tested (dissociation constants (pKa) equal 1.94 and 6.22), using the same chemical compounds; (ii) is not consumed by *Y. lipolytica* (as determined by HPLC analysis); (iii) does not hamper growth, if applied at selected molarities; (iv) does not impose osmotic stress, when used at higher/different molarity (within 250 mOsm kg^−1^; below the stress level for *Y. lipolytica* [56]); and (v) has sufficient buffering capacity at adopted concentrations (Fig. 2a, b),
ii. The adopted temperature range supports growth and provides differentiation in the r-Prots synthesis level **(**Fig. 2c),
iii. To impose an actual OA stress, but still—support growth and r-Prot synthesis, the headspace volume exchange must be minimized to 0.004 mL min^−1^ **(**Fig. 2d),
iv. Provision of a higher nitrogen source (15 g L^−1^) is necessary to enable more complete carbon utilization and full phenotype development within a reasonable time frame (48 h) (Fig. 2e),
v. Cubic geometry of the cultivation vessels and larger volume of the culture enables faster and more pronounced phenotype development; comparison of readouts from the outer and from the inner wells of 24-well round (1 mL) and 24-well square (2.5 mL) plates, revealed that the well-to-well variation is significant for the former, and insignificant for the latter (p-value < 0.05); the squared wells enabled more homogenous mixing during cultivation and no biomass attachment to the walls of the well; using 24-deep-well square plates enables maintaining high-throughput character of the screen and reliable phenotypes assessment **(**Fig. 2f), and
vi. Carbon source at > 30 g L^−1^ is sufficient to support growth at the nitrogen concentration of 15 g L^−1^, and is not fully consumed by the end of the culturing time (does not impose additional, uncontrolled starvation condition) (Fig. 2g).

With the pre-optimized and validated protocol for high-throughput screens of *Y. lipolytica* in our hands, we developed an experimental design that would enable systematic investigations into the effects induced by the variables and their combinations. The design rendered 104 culture runs to be implemented for each of the 125 strains and the controls (Table 1). The levels of the variables were encoded, as depicted in Fig. 1. Acquired data on the strains’ growth, synthesis of the reporter r-Prot, or its normalized measure (r-Prot normalized per biomass) were carefully curated, and subjected to statistical significance analysis and mathematical modeling, according to “Data processing and statistical analyses” section.

### YaliFunTome enables the withdrawal of biological sense from math

#### Navigation through YaliFunTome

The amount and informativeness of the data we gathered from completing our experimental design prompted us to present them in the form of a searchable catalog YaliFunTome (*Yarrowia lipolytica* Functional Screens of Transcription Factor-ome Database: https://sparrow.up.poznan.pl/tsdatabase/), to facilitate the withdrawal of biological sense from extensive sets of numerical data. In this, we were inspired by our Colleagues, who put a great effort into preparing and curating publicly open databases, such as: mentioned above Yeastract + , which enables predictions of efficient genetic engineering strategies, and its extension—PathoYeastract, enabling the analysis and prediction of transcription regulatory associations in the pathogenic yeasts [57], y-mtPTM, that provides a comprehensive list of experimentally-validated mitochondrial post-translational modifications [58], or YeastRGB, merging, processing and standardizing graphical data on protein abundance and subcellular localization [59].

Navigation through the YaliFunTome database should start from the ‘readme’ tab, which contains all the necessary information to guide the user throughout the database. The ‘readme’ section is divided into four subsections: (i) ‘general information’ about the project; (ii) ‘experimental setup’ describing how the project was technically implemented; a must-see-before-starting tab; (iii) ‘data presentation’, illustrating how to interpret records of the database search; and the fourth tab showing Response Surface Methodology (RSM) models for the; and (iv) ‘control strain’, which were used as a reference to calculate Fold Change (FC) data for TF-OE-ing strains.

The ‘global view ‘ tab directs the user to three ‘global heat maps’ with FC values for all the TF-OE-ing strains gathered in collective plots. The three heat maps show the data on each of the three analyzed parameters: ‘growth’, ‘total r-Prot’, and ‘normalized r-Prot’, separately. Data within the table are color-coded according to the legend provided on top of the subpage, and are interactive: (i) while hoovering over a specific color-coded cell, a short note appears summarizing the conditions under which the response was elicited, its numerical value, and whether the response was statistically significant at p < 0.05; and (ii) while clicking on a specific ‘Yali number’, the user is directed to a specific TF-OE strain’s subpage.

The ‘search’ tab links the user to search tools, enabling two modes of querying the database: (i) based on ‘Yali number’, and (ii) ‘Condition-responsive TF’. The former can be used to characterize the functional behavior of a specific TF across an array of conditions, while the latter—to screen for TFs that were responsive to a specific combination of environmental factors, implemented in the experimental design.

The ‘cite’ tab directs to this article’s citation and contact details. More details about querying the database and the retrieved records can be found in “Querying YaliFunTome” and “YaliFunTome record’s content” sections.

#### Querying YaliFunTome

Browsing the YaliFunTome database is enabled in two modes: (i) ‘Yali number’; and (ii) ‘Condition-responsive TF’.

The ‘Yali number’ search enables browsing the database based on specific Yali numbers of genes, according to the naming convention from the first release of the *Y. lipolytica* genome [13] (listed in Additional file 2: Table S1). A specific Yali number can be selected from a collapsible drop-down list. The search record directs the user to three links corresponding to the set of heat maps showing FC in the read parameter for a specific TF-OE-ing strain (‘growth’, ‘total r-Prot’, or ‘normalized r-Prot’). Clicking on the selected parameter gets the user to a Yali number’s specific subpage, which consists of (to be found by scrolling down):

i. A header providing YALI name, number of TF in the experimental design, assigned name (if available), gene identifier, and links to NCBI, RefSeq DNA, RefSeq Peptide, and UniProtKB,
ii. Three heat-maps (for ‘growth’, ‘total r-Prot’, or ‘normalized r-Prot’), arranged according to Fig. 1a, and factor’s contribution tables encoded as presented in Fig. 1b, c. The system of data presentation and variables’ coding is reminded at the top of the subpage, for the user’s convenience; both the heat maps and the Factor’s Contribution tables are interactive, providing the user with detailed information about a specific response point, upon hovering over it.

The ‘condition-responsive TF’ search mode requires first defining the search constraints. The user can browse the database based on an elicited response in one of three measured parameters (‘growth’, ‘total r-Prot’, or ‘normalized r-Prot’), which could be: (i) ‘up’—higher in the TF-OE-ing strain compared to control (FC > 1.0 in a specified point); (ii)’down’ (FC < 1.0); or show (iii) ‘inverted phenotype’, so the opposite FC response under extreme levels of a variable by a single TF-OE-ing strain (e.g. FC > 1.0 in pH 3.0 and FC < 1.0 in pH 7.0). These response constraints are set in combination with one of the studied variables (pH, temp, carbon source, nitrogen source, or OA). The last drop list specifies the constraining level of the searched variable, or specifies the levels of the variable compared in the ‘inverted phenotype’ search. The output page returns Yali numbers of the TFs that elicited a statistically significant response, meeting the entered constraints, and an additional 10% level of difference in FC between the TF-OE and the control strains. Yali numbers are sorted by the highest FC values for the ‘up’ search, by the lowest—‘down’ search, or by the highest delta of FC values between extreme environmental conditions for the ‘inverted phenotype’ search. The records specifying TF numbers that were responsive are collapsible—after expanding, the list of specific combinations of variables under which the query conditions were met is listed.

#### YaliFunTome record’s content

The results from the experimental plan may reflect ‘growth’, ‘total r-Prot’, or ‘normalized r-Prot’ by the TF-OE-ing strain and the control strains. The principal mode of data presentation is an FC of a given measure read for the TF-OE-ing strain over the control strain. The data for each TF-OE-ing strain were arranged according to the variables combinations scheme in Fig. 1a, and color-coded to facilitate simple interpretation. The raw data for the three measured parameters were first used to generate RS models. Such models were shown in the ‘control strain’ tab for the control strain. The same set of models was prepared for all the TF-OE-ing strains (not shown). Modeling the data for TF-OE strains and the control strains enabled the estimation of the response within each point covered by the RS plot (within the adopted ranges of the variables). In addition, the models allowed the assessment of the variables’ contribution (individually or as interactions; the latter not shown) to the respective measured parameter. These individual variables’ contribution is quantitatively expressed (as percentage values) in the Factor's Contribution ranking tables, provided on each TF-OE-ing strain’s subpage (Fig. 1c). Both the heat maps and the factor contribution tables were color-coded according to the provided legends. Details on the data presentation scheme can be found in the ‘read me’—> ‘data presentation’ tab in YaliFunTome database.

### YaliFunTome in practice—case-studies

#### Klf1 (YALI0D05041g) is a newly identified general booster of r-Prots synthesis

By simple inspection of the ‘global view’ plots for ‘growth’, ‘total r-Prot’, or ‘normalized r-Prot’ it is possible to fish out phenotypes eliciting strong responses nearly irrespectively from the environmental variables.

**Fig. 3:**
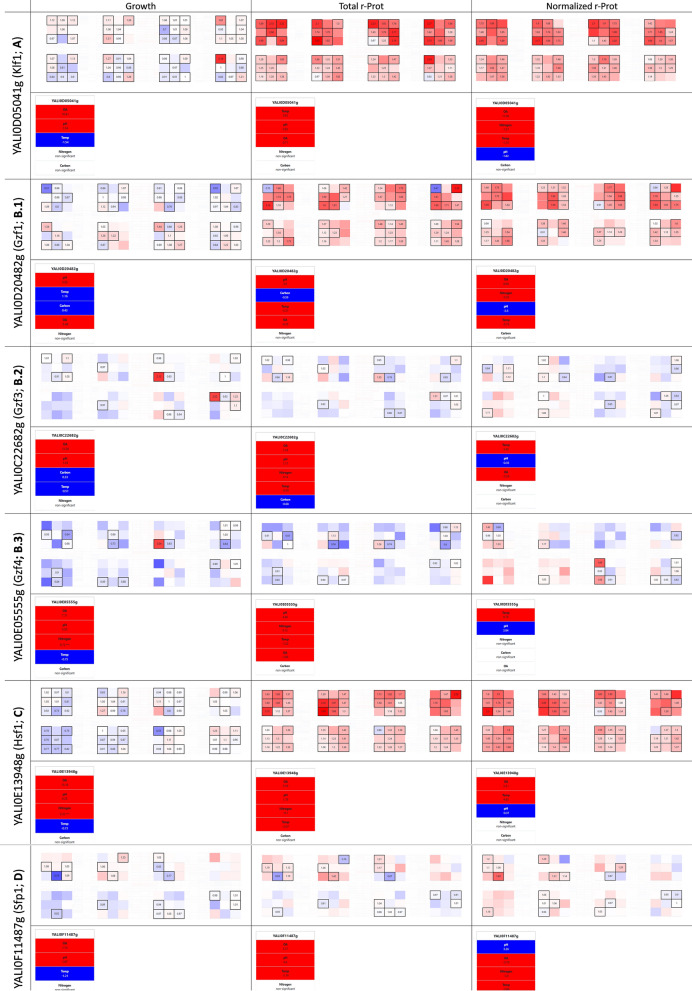
Selected phenotypes from the YaliFunTome database—the enhancers of r-Prots synthesis. Selected phenotypes are presented according to the data presentation format (Fig. 2). YALI number and assigned name (if available) are given in the first column. The following columns present heat maps and factor’s contribution rankings in terms of growth, r-Prots synthesis, and normalized measure of r-Prots synthesis

Such a pronounced phenotype was observed for a strain OE-ing YALI0D05041g gene, encoding a Zinc finger TF Krueppel-like factor 15 (Klf1; TF126). According to NCBI’s conserved domain search, *Y. lipolytica*’s Klf1 bears two C2H2 Zinc finger domains at the N-terminus [60], studied this specific gene as one of the model transcripts for the intron retention phenomenon in *Y. lipolytica*. Klf1’s mRNA was classified as one of the transcripts displaying intron retention but at a low level. Online translation prediction of the unspliced transcript shows that the intron retention results in premature stop codon occurrence, so it cannot be functional as an alternatively spliced variant, that would render an additional domain, depending on the signals. In yeast, Klf1 was extensively studied in the context of long-term G0 quiescence induced by nitrogen starvation [61]. As shown in those studies on *Schizosaccharomyces pombe*, the expression level of Klf1 in G0 (no nitrogen) was higher than in vegetative growth, when nitrogen was available. At the physiological level, the ∆*klf1* mutants exhibited significantly enlarged cell volume, they were metabolically active but unable to restore cell division. Prolonged nitrogen starvation of the ∆*klf1* mutants triggered a fatal failure in the cell’s morphology maintenance—accumulation of chitin-like material caused severe deformation of the cytoplasm and cell shape. Transcriptomic analyses demonstrated that Klf1 directly regulates genes implicated in cell wall renewal, oxidative stress response, glycolysis, nutrient uptake, RNA-mediated chromatin silencing, glycosidation, and methylation [61]. In higher Eukarya, Klf1 was shown to promote longevity by combating oxidative stress [62]. It was demonstrated that Klf1 relocalizes to the nucleus following a mild ROS pulse. In those studies, the oxidative stress originated from mitochondria, followed by xenobiotics treatment. Transcriptomic analysis revealed that the response elicited by Klf1 is more elaborated rather than simple combating oxidative stress. In the context of our studies, we found out that *KLF1*’s OE in *Y. lipolytica* greatly promotes r-Prots synthesis capacity, nearly irrespectively of the adopted variables’ combination (Fig. 3a). The enhancement expressed in FC of (normalized) r-Prots synthesis was more pronounced under high OA (contribution of high OA was positive and higher than in the control strain by 11.08%), frequently reaching ≥ 2.0 FC. The promoting phenotype was enhanced under organic nitrogen provision, higher temperature, and lower pH; and was independent from the carbon source provided. The role of Klf1 has not been studied in *Y. lipolytica* to date. Considering the commonly assigned to Klf1 implication in stress response, and our previous studies on the effects triggered by over-synthesis of r-Prots in *Y. lipolytica* [63], it is tempting to state that the increased abundance of Klf1 in combination with ER-derived oxidative stress (caused by r-Prots over-synthesis) were the two factors enabling the display of the Klf1’s promoting phenotype. As shown by our auxiliary experiments (to be published), knock-out in *KLF1*’s loci did not trigger any significant change to r-Prot synthesis capacity in *Y. lipolytica* (under ‘optimal’ conditions – a central point of the design; Additional file 1: Fig. S1a), which suggests some level of redundancy within this scope with some other factors.

#### Gzf1 (YALI0D20482g) is the only ‘global r-Prots enhancer’ from among tested genuine GATA-binding zinc finger family representatives (Gzf1, Gzf3, Gzf4)

Other ‘global’ r-Prots synthesis enhancers identified through the ‘global view’ plot inspection comprised Gzf1 (YALI0D20482g; TF037) and Hsf1 (YALI0E13948g; TF068). The former belongs to a six-member family of GATA-binding zinc finger TFs, *i.a.* involved in nitrogen catabolite repression (NCR). Gzf1 was studied in *Y. lipolytica,* along with the other three genuine representatives of the Gzf family, in the context of NCR’s role in lipid accumulation regulation [64]. While investigating the molecular background of the nitrogen provision-lipid accumulation interplay, the Authors functionally characterized the Gzfs in *Y. lipolytica*. Insightful sequence-structure analysis demonstrated that Gzf1, so the current ‘global r-Prots synthesis enhancer’, is highly similar to homologs from *Aspergillus nidulans* and *Neurospora crassa* activated in response to nitrogen starvation, and is likely to operate as an activator of NCR genes, rather than a repressor (like Gzf3; YALI0C22682g). Our current studies demonstrated that upon its OE, Gzf1 greatly enhances the synthesis of r-Prots in *Y. lipolytica* (total and normalized measures; Fig. 3b1). This enhancing phenotype is promoted under high OA (factor’s contribution to ‘normalized r-Prots’ is higher by 9.69% than in the control), organic nitrogen, higher temperature, and lower pH. Factor’s contribution analyses demonstrated that the nitrogen source variable did not exert any significant contribution to the growth (and total r-Prots synthesis) in the *GZF1*-OE-ing strain. Considering its putative role as an NCR activator, this could be attributed to enhanced nitrogen scavenging capacity when inorganic nitrogen was provided. However, nitrogen was also a non-significant variable for the control strain’s growth. Consistently, in the previous studies, it was observed that *GZF1* was not essential for growth on simple nitrogen sources (as was Gzf2; YALI0F17886g not covered by the YaliFunTome) [64]; even though it was the most upregulated gene from the Gzfs when an organic nitrogen source was changed to inorganic. A similar transcriptional response was also observed for *GZF4* [64]; altogether suggesting that Gzf2 (inducer) and Gzf3 (suppressor) are the overriding nitrogen assimilation regulators, while Gzf1 and Gzf4 serve as ‘rapid-reaction force’ of this regulation. Our results demonstrate the OE of neither *GZF3* (Fig. 3b2) nor *GZF4* (YALI0E05555g; Fig. 3b3) exert any direct positive effect on r-Prots synthesis in *Y. lipolytica*. While it seems understandable for the NCR’s repressor Gzf3, it remains to be clarified for the Gzf4, whose specific role in NCR has not been determined [64]. The YaliFunTome database’s ‘global r-Prots enhancer’, Gzf1, was previously identified as a promoter of r-Prots synthesis in *Y. lipolytica* upon its OE, but under a different array of variables [39]. Our auxiliary studies on *Δgzf1* genotype (Additional file 1: Fig. S1a) showed that it displays no effect on r-Prots synthesis [39], consistent with the results by [64]. Only its OE elicited the promoting phenotype; as was found for Klf1 (mentioned above). Such a consistency supports the generalizable character of these findings.

#### Hsf1 (YALI0E13948g) specifically promotes protein synthesis at the expense of growth

*HSF1* OE elicited a similar promoting response in terms of r-Prots synthesis as Klf1 and Gzf1, however, it acted more selectively in this regard (Fig. 3c). It promoted the synthesis of proteins even at the expense of growth under inorganic nitrogen provision (r-Prot up, when growth is down). To our interpretation, the significantly enhanced r-Prots synthesis under *HSF1* OE leads to a higher demand for resources. Specifically, based on the results of the factor’s contribution analysis, we speculate that nitrogen became limiting, as this specific variable gained significance for growth upon *HSF1* OE (not significant for *KLF1*-OE, *GZF1*-OE, and the control strain). The possible shortage in nitrogen supply prompted cell cycle arrest and growth inhibition, while the protein synthesis was still ongoing. Such a type of interplay between intensive r-Prots synthesis and growth was quantitatively expressed in our previous studies [65]. Now, based on the developed mathematical models we show that within the range of tested conditions, nitrogen became the limiting factor under intensive r-Prots synthesis.

A decreased correlation of growth and r-Prot synthesis observed for *HSF1*-OE-ing strain (Pearson coefficient between FC values for ‘growth’ and ‘total r-Prots’ r < 0.45) was not observed for the other TFs promoting r-Prot synthesis, like Klf1 or Sfp1 (discussed below). For the latter, the FC values for total r-Prots and growth were more positively correlated (r > 0.58). The positive correlation between the two parameters indicates that the two biological processes do not compete but rather act synergistically. Considering that the reporter r-Prot used here acted as a gauge of total protein synthesis capacity, such a synergistic action is natural and expected—since high protein synthesis is a prerequisite for robust growth.

The promoting role of Hsf1 in r-Prots synthesis seems straightforward to interpret. Hsf1 serves as the major activator of the cell’s folding and chaperoning capacity, and induces the cytoplasmic form of UPR [66]. It also acts as the global regulator of the general stress response, which activates multiple biological processes promoting r-Prots synthesis [7]. The beneficial effect of *HSF1*’s OE on r-Prots synthesis in *Y. lipolytica* was also observed previously, under the infliction of a different set of variables [39].

#### Ribosome biogenesis-associated Sfp1 (YALI0F11487g)—‘a fussy eater’

A strong promoting effect on growth and r-Prots synthesis was expected from the strain OE-ing an *SFP1* gene (YALI0F11487g; Split Finger Protein; TF123). Sfp1 is a stress- and nutrient-sensitive regulator of ribosomal protein gene expression, localized to the nucleus in exponentially dividing cells [67]. Sfp1 was recognized as a downstream effector of the TOR pathway (in part through the PKA pathway), which is inactivated under stress or nutrient limitation; under such conditions, Sfp1 is relocalized through nucleocytoplasmic trafficking to the cytoplasm, and the expression of ribosomal protein genes is down-regulated. In this sense, Sfp1 localization is regulated in a manner opposite to that of the known TOR downstream transcriptional effectors (Msn2/4, Gln3, Gat1, or Rtg1/3) activating stress-response programs. Considering its mode of operation, we expected that an increased abundance of Sfp1 would contribute to significantly enhanced growth and r-Prots synthesis under favorable environmental conditions. Indeed, we observed some enhancement in growth under selected variables combined with high OA; and a corresponding increase in r-Prots synthesis (Fig. 3d). However, the level of FC values either for growth or r-Prots was rather disappointing. Considering the abovementioned pattern of Sfp1 relocalization in response to different stimuli (including the phase of growth), we hypothesize that the sampling time, which was optimized to enable standardized reading of the majority of phenotypes from the collection, was, in this specific case, inadequate. Phenotype reading was conducted at 48 h of culturing—by that time, all the strains reached a stationary phase of growth, but nutrients were not yet limiting. As demonstrated for *S. cerevisiae* [67], the growth rate cessation in the stationary phase is accompanied by cytoplasmic localization of Sfp1, and inactivation of its transcriptional program. Experiments testing this hypothesis are also currently underway.

#### Azf1 (YALI0A16841g) and Dep1 (YALI0F05896g) act specifically against r-Prots synthesis

**Fig. 4:**
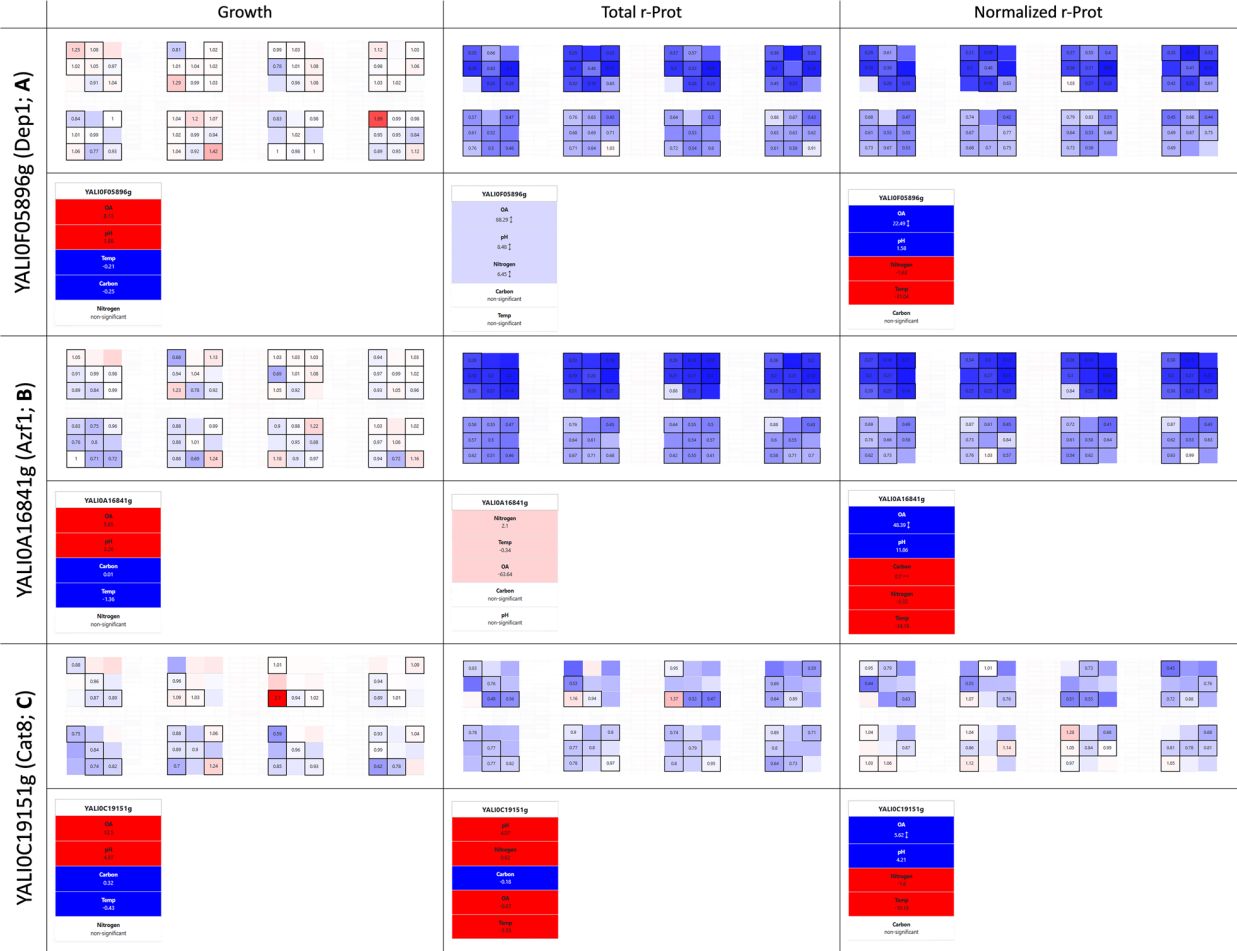
Selected phenotypes from the YaliFunTome database—the repressors of r-Prots synthesis. Legend as in Fig. 3

Browsing YaliFunTome database revealed the existence of specific silencers of r-Prots synthesis, in opposition to the abovementioned global enhancers. Such global silencers specific to r-Prots synthesis are characterized by not affected growth (mean FC: 0.96–1.01), but severely decreased r-Prots synthesis capacity (mean FC: 0.4–0.49). Among these, we identified Dep1 (YALI0F05896g; Deregulated Expression of Phospholipid biosynthesis), whose OE drastically reduced the accumulation of the reporter r-Prot (Fig. 4a). Previously, Dep1 was found to enhance the accumulation of lipids in *Y. lipolytica* [37], and specifically—activate phospholipid biosynthesis in *Fusarium* sp. [68]. In contrast, [69] identified Dep1 as a repressor of phospholipids synthesis genes (e.g. *INO1, CHO1, OPI3*) in *S. cerevisiae*. Our auxiliary experiments demonstrated that the *Δdep1* strain displays an inverted phenotype in terms of r-Prots synthesis—enhances it by ~ threefold (‘optimal’ conditions; Additional file 1: Fig. S1b). Such behavior suggests its direct implication in the regulation of this process.

This kind of direct interaction was not observed for the second identified ‘global silencer’ of r-Prot synthesis, Azf1 (YALI0A16841g; Asparagine-rich Zinc Finger protein). While its OE triggered a dramatic decrease in r-Prots synthesis (Fig. 4.b), *Δazf1* mutation rendered no aberrant phenotype in this regard (‘optimal’ conditions; Additional file 1: Fig. S1b). According to SGD (*Saccharomyces* Genome Database), Azf1 is a carbon source-responsive TF, and in the presence of glucose, it activates genes involved in growth, carbon metabolism, and invasive growth. Indeed, we observed that the *AZF1*-OE strain displayed enhanced filamentation when compared to the control strain (Additional file 1: Fig. S2), and that the ‘carbon’ variable had a significant contribution to the developed phenotype (dominant level − 1, glucose; Fig. 4b).

Notably, for both the ‘global silencers’ we noticed that the OA variable’s direction of contribution to r-Prots synthesis was inverted when compared to the control strain. Typically, high OA was the key variable with the highest promoting impact on the (normalized) r-Prot synthesis; practically meaning that high OA (even if normalized per control strain that also synthetizes r-Prot) still has the highest importance in shaping the response. For Dep1- and Azf1-driven response, it was the low OA, that promoted the limiting phenotype display (the higher OA the lower the ‘normalized r-Prot’ value). Similarly, such an ‘inverted phenotype’ in response to OA was observed for a strain OE-ing *CAT8* gene (YALI0C19151g; CATabolite repression; Fig. 4c), whose OE also triggered a decrease in r-Prots synthesis, particularly marked under high OA. Cat8 is a Zinc cluster transcriptional activator, binding to carbon source-responsive elements in the absence of glucose (contrary to Mig1). Although the Cat8-induced phenotype was not as radical as in the case of Azf1 or Dep1 [growth mean FC: 0.96; (normalized) r-Prot mean FC: (0.82) 0.76], it still justified its classification as a ‘global silencer’. Its known involvement in the regulation of gluconeogenesis and the glyoxylate cycle makes it functionally similar to the two others, suggesting the operation of a general pattern: activation of carbon anabolism genes specifically limits protein synthesis in a categoric manner.

#### Unmaking anoxia a problem for *Y. lipolytica*

As mentioned above, insufficient OA is the key technical limitation in industrial bioprocesses implementing *Y. lipolytica*. Its strictly aerobic metabolism, relying on the TCA cycle and β-oxidation, forces high oxygen provision for full development of phenotype. Apart from the implementation of technical bioprocessing solutions aiding higher oxygen provision, some efforts to minimize the high requirement for oxygen by genetic manipulations have been made. One such interesting approach comprised the OE of bacterial hemoglobin [47, 70], which ultimately led to the enhancement of r-Prot and erythritol synthesis.

**Fig. 5:**
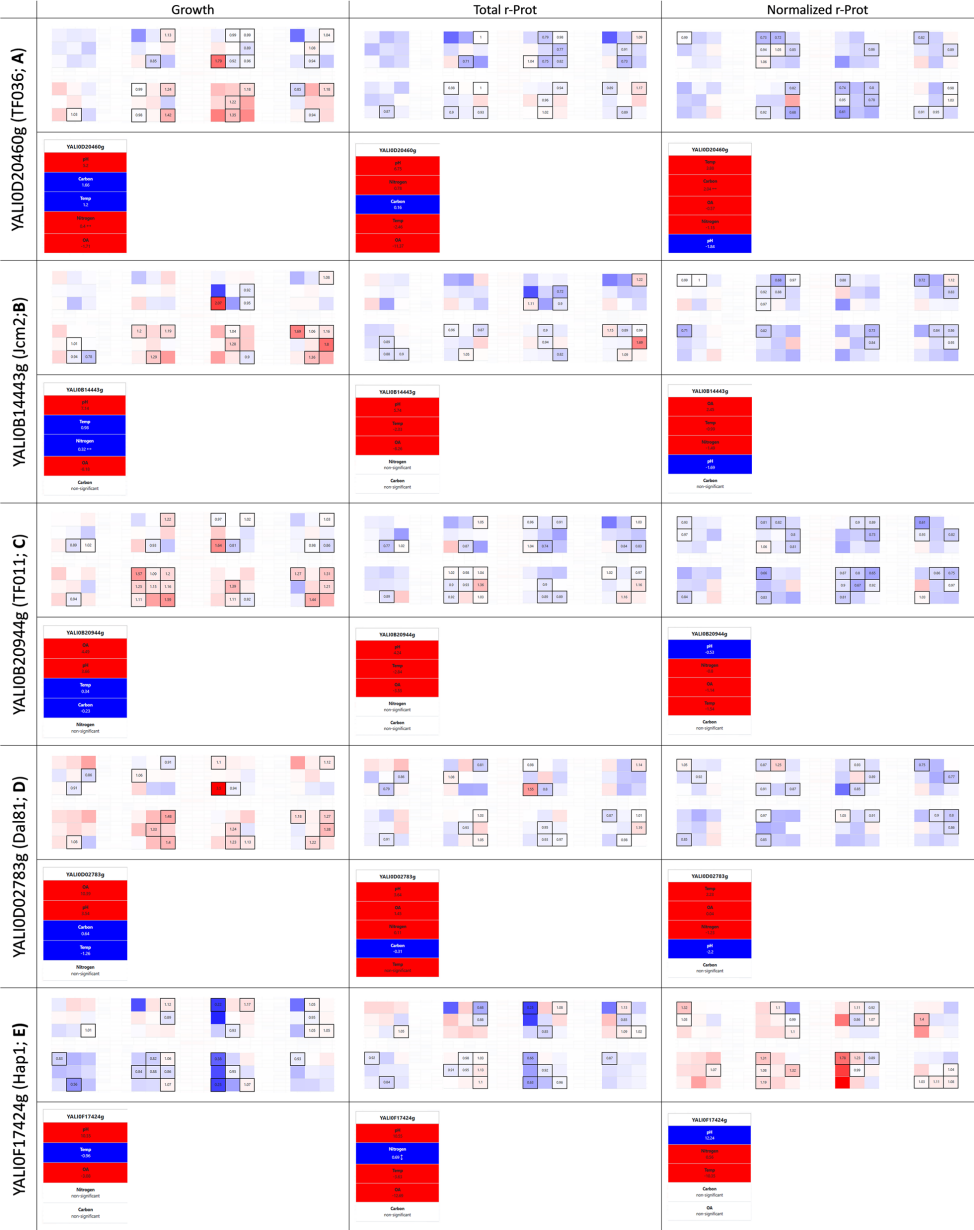
Selected phenotypes from the YaliFunTome database—the OA-responsive TFs. Legend as in Fig. 3

Our methodological approach followed by browsing the YaliFunTome database enabled the identification of TFs that displayed significantly enhanced growth (compared to the control strain) when the OA limitation was inflicted (Fig. 5a–d). These were TF036 (YALI0D20460g; contribution of low OA higher by 14.6% than in the control, calculated by modeling FC data), Jmc2 (YALI0B14443g; TF009; 10.6%), TF011 (YALI0B20944g; 7.3%), and Dal81 (YALI0D02783g; TF118; 2.7%) (Additional file 1: Fig. S3). All the TFs shared the same pattern for averaged responses for enhanced growth (FC: 1.06–1.14), and a slightly inhibitory effect on r-Prots synthesis (FC: 0.91–0.97). The highest FC gain in growth under low OA reached even 60–80% under OE of these TFs. Except for Dal81, all these TFs contributed to significant limitations in total r-Prots synthesis, typically decreased by 10–20%, even under conditions promoting growth (FC for growth up, while FC for r-Prots down). The function for these TFs in *Y. lipolytica* has not been assigned yet. A blastp search (under default settings) showed that TF036 shares some similarities with a positive regulator of lactose-galactose metabolism, Jmc2 is similar to lysine-specific methylase, TF011 is similar solely to the other putative proteins from *Y. lipolytica*, while TF118 shows similarity to Dal81 TF. Recently, Jmc2 has been identified as one of the upregulated genes in *Y. lipolytica* evolved strain exhibiting enhanced growth and lipid accumulation [71]. In that study, it was defined as a representative of JmjC domain family of histone demethylase which promotes global demethylation of H3K4 subunit. According to SGD, Dal81 (Degradation of Allantoin) is a positive regulator of genes in multiple nitrogen degradation pathways; however detailed studies demonstrated that the TF most probably does not bind to the specific motif in the promoter region of many genes involved in allantoin catabolism. The current lack of solid evidence for the TFs’ putative functions disallows any speculations on the possible mechanisms by which the enhanced growth under anoxia is achieved. Considering the significance of practical implications of identifying ‘anoxia-resistance factors’ in *Y. lipolytica*, further functional studies are underway.

In strong contrast to the effects triggered by the TFs enhancing growth under low OA, we identified such that make *Y. lipolytica* significantly more sensitive to limited OA (Fig. 5e). As summarized in SGD, Hap1 (YALI0F17424g; TF120; Heme Activating Protein; previously referred to as CYP1) is known to be involved in the transcriptional regulation in response to OA using its ability to bind heme as a proxy for detecting oxygen levels. It is localized in the nucleus and mitochondrion, and can act as both – activator and repressor of transcription [10]. Under high OA, Hap1 activates the genes involved in respiration, cell cycle progression, and ROS defense, promoting growth and respiratory metabolism in *S. cerevisiae* [10]. While the mechanisms of oxygen sensing and heme signaling in *Y. lipolytica* have not been investigated to date, our current results strongly suggest that the YlHap1 homolog is involved in such regulation (Fig. 5e). Our data demonstrate a very strong limitation of growth upon a combination of *HAP1*-OE and low OA. In previous studies with *S. cerevisiae*, OE of *HAP1* triggered a significant increase in the r-Prots synthesis capacity [11]. Insight into Hap1’s regulome revealed that it activates a set of oxidative stress response genes, therefore mitigating the negative effect of ROS accumulation associated with protein folding. In our current studies, the total r-Prot synthesis was not enhanced, however, when normalized per growth, the r-Prots synthesis capacity of *Y. lipolytica* was enhanced by an average value of 14% (Fig. 5e). That increase was mainly observed under low OA, hallmarking that the growth limitation under this condition was at least partly decoupled from r-Prots synthesis (that was enhanced). Interestingly, we observed a highly marked contribution of the pH variable to growth and r-Prot synthesis by the *HAP1*-OE-ing strain. That strain exhibited a strong growth limitation under acidic pH. Hap1’s implication in pH response regulation has not been studied to date, in contrast to the evidenced functional operation of the Rim101-governed pathway [72]. Hence, the determination of whether the action of Hap1 is an actual causative factor of the decreased growth under pH 3, remains to be addressed.

#### What about the golden standards for enhancing stress resistance—Mhy1, Msn4?

**Fig. 6:**
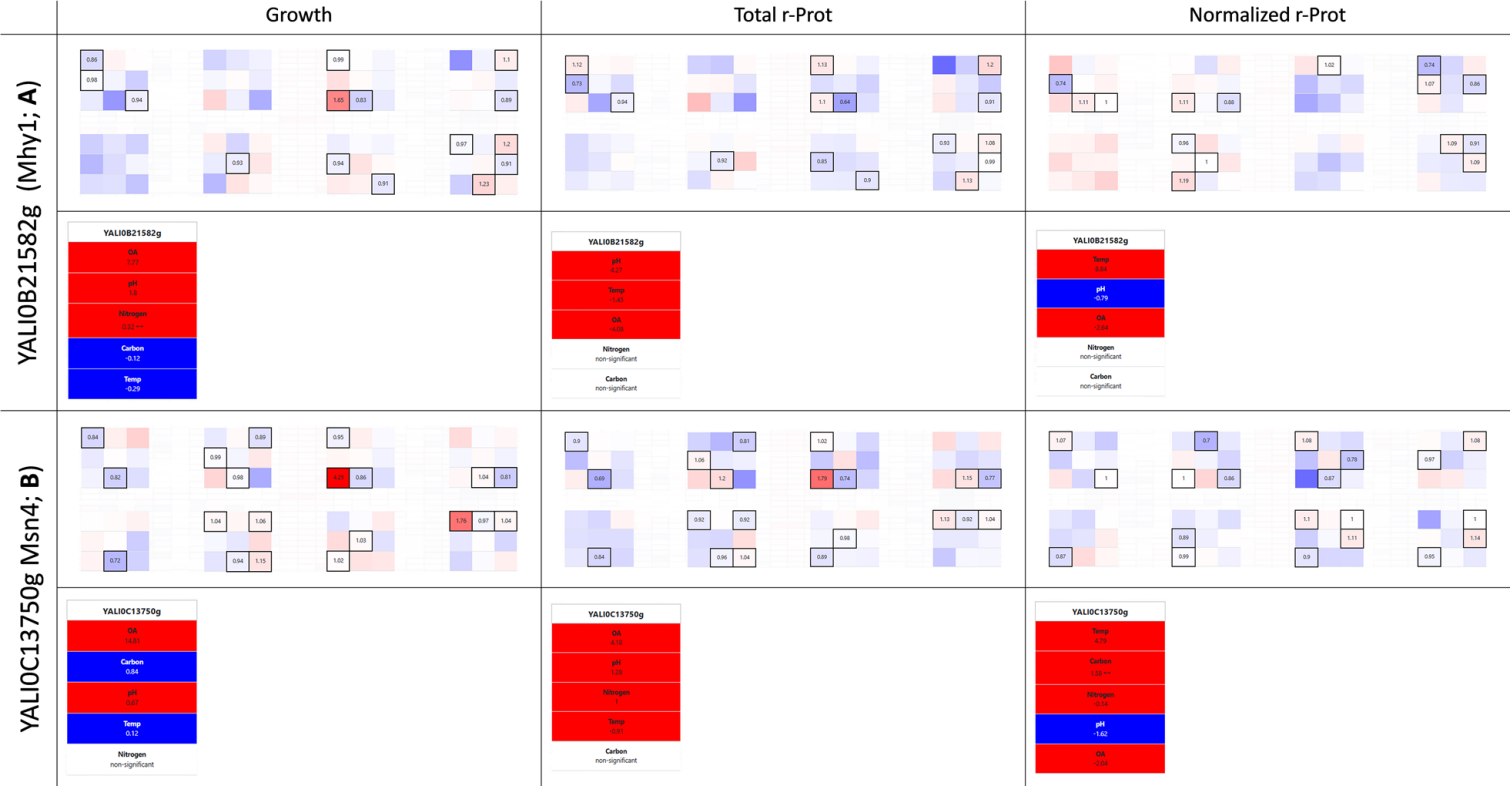
Selected phenotypes from the YaliFunTome database—the global stress response TFs. Legend as in Fig. 3

In pursuance of stress-resistant yeast phenotypes, engineering one of the general stress response factors is frequently applied. These comprise the Msn2/4 zinc finger TFs family that typically binds to cis-acting DNA stress response elements (STRE). In *Y. lipolytica*, two such Msn2/4 family representatives are known and were studied in this context—Mhy1 (YALI0B21582g; TF095) and Msn4 (YALI0C13750g; TF107). *MHY1*’s expression was shown to be dramatically increased in *Y. lipolytica* during the yeast-to-hypha transition, neutral-alkaline pH, and presence of glucose [27, 73]. Mhy1 was indicated as a key positive regulator of both alkaline- and glucose-induced filamentation, inhibited in the presence of glycerol [73]. Hence, in our studies, we expected to observe a strong contribution of these variables to the growth of the *MHY1*-OE-ing strain (Fig. 6a). Indeed, a significant contribution of glucose and a higher pH to the growth of this strain was observed; in addition, to an increased frequency of filament occurrence (Additional file 1: Fig. S2). However, the strain’s growth was not promoted (average FC: 0.96), even though some of the inflicted conditions can definitely be considered stressful/promoting Mhy1’s action. In high relevance to this observation, [28] evidenced recently that Mhy1 action is not required for increased stress resistance in this species but plays a role in the dimorphic transition. The lack of Mhy1’s implication in stress response was confirmed in the following studies [74]; which agrees well with our current observations.

The other representative of the Msn2/4-like TFs family in *Y. lipolytica*, Msn4 was previously shown to serve as a regulator of acid-induced stress [28]. In our studies, this effect upon *MSN4*’s OE was rather moderate (Fig. 6b); although leading to some very high FC in growth (4.25), but in isolated cases. Globally, *MSN4*-OE contributed to minor induction of growth under the adopted conditions (average FC: 1.04). Previously, *Y. lipolytica* strains deleted in *MSN4* loci displayed growth defects when grown at pH 3, while its OE impacted the cells’ morphology—reduced cell chain formation in wild-type cells but did not affect cell size [28] (compare Additional file 1: Fig. S2).

For both the Msn2/4-like TFs family, the effect of their OE on r-Prots synthesis was relatively small, and not demonstrating any noticeable pattern. It was rather disappointing considering the recent success of [12], who by OE-ing *MSN4* in *K. phaffi* significantly improved r-Prot synthesis and secretion (from 52% in small-scale cultures, up to > 300% when co-OE with other factors and in bioreactor cultivations).

#### Counter-intuitive effector acting on r-Prots synthesis in inorganic nitrogen—Hoy1 (YALI0A18469g)

Organic nitrogen, high OA, and mild acidic to neutral pH are the variables expected to promote growth and r-Prot synthesis. Considering that sufficient OA supply is the key technical limitation in the bioprocesses employing *Y. lipolytica*, it was exciting to discover TFs that promote growth under limited oxygen provision (“Unmaking anoxia a problem for *Y. lipolytica*” section). Likewise, considering that the cost of complex, organic nitrogen source constituted the highest unit production cost in the pilot-scale waste-free process of r-Prot production [49], it would be exciting to identify a factor that would enable enhanced r-Prot synthesis in inorganic nitrogen. Our current data suggest the appearance of such a tendency upon OE of the *HOY1* gene (YALI0A18469g; TF099; Homeobox-containing gene). In *Y. lipolytica* Hoy1 plays a role in the morphological transition and is not essential for growth, but its OE was proved to promote hyphal growth [26]. Based on its promoter sequence architecture, *HOY1* was proposed to be deregulated in response to stress cues, and downstream of signals for amino acid biosynthesis regulation/nitrogen starvation. In fact, this co-occurrence of stress response and nitrogen regulation cis-elements well corresponds with the following findings by Szabo and Štofaníková 2002. It is known that *Y. lipolytica* grows in filamentous morphotype at neutral-alkaline pH, while acidic pH promotes growth in ovoid form. That study evidenced that the pH-dependent morphogenetic shift operates solely in the presence of an organic nitrogen source, and is still operable in *Δrim101* background (the pH sensing and signaling pathway is off). Thus, the Authors concluded that pH affects the formation of hyphae indirectly—by modulation of availability and/or utilization of transportable nitrogen sources, and not by a Rim101-governed, pH-response mechanism. The combination of data from those two studies portrays Hoy1 as a TF whose expression is enhanced under a dimorphic transition that is natively activated in the presence of organic nitrogen.

**Fig. 7:**
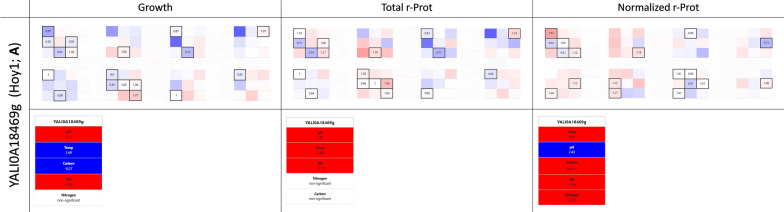
Selected phenotype from the YaliFunTome database—the nitrogen-responsive TF. Legend as in Fig. 3

In our experiments, we synthetically enhanced the abundance of TFs under conditions that natively do not promote their expression. Considering what is known about *Y. lipolytica*’s Hoy1, its expression should be attenuated under inorganic nitrogen source provision. Strikingly, as depicted in (Fig. 7a), its artificially forced presence under these conditions contributed to enhancement in the r-Prots synthesis. Taking into account findings by Szabo and Štofaníková 2002 it is highly plausible that the ‘synthetic presence’ of Hoy1 facilitated nitrogen capturing under its scarcity; which in turn contributed to enhanced r-Prots synthesis. Indeed, as demonstrated by our factor’s contribution analysis, nitrogen was a variable that gained significance in terms of normalized r-Prots synthesis measure for the *HOY1*-OE-ing strain. The importance of the organic nitrogen source presence was decreased when compared to the control strain; and when the models were developed on FC values, the presence of an inorganic nitrogen source (level − 1) was a prerequisite for the enhanced r-Prot synthesis capacity phenotype display (the factor contribution higher by > 7%) (Additional file 1: Fig. S3).

We consider the finding of Hoy1’s promoting action in the context of r-Prot synthesis from inorganic nitrogen an important discovery of high applicatory potential.

## Conclusions

Without extensive insight into the molecular biology of a cell, one can intuitively state that modification of complex traits, like stress resistance or r-Prots synthesis, will be more adequately addressed using ‘global transcription machinery engineering’. Such an approach fine-tunes multiple molecular identities according to a nature-designed program to elicit a specific response. It is the investigator’s work to define, which of the programs most adequately corresponds with the target cellular functionality, and hijack it.

In our research, we made an effort to screen a repertoire of the transcriptional programs developed over the evolution of *Y. lipolytica* species. A similar high-throughput study into TFs’ action was earlier executed for *S. cerevisiae* [67]; where a collection of > 200 strains OE-ing GFP-fused TFs was investigated for the changes in TF’s localization as a proxy of its activation. While that former study aimed at providing evidence on the TFs’ activation status, in our research we put great effort into finding the conditions that prompt the TF-driven program’s onset, by challenging the strains with an array of variables and their combinations. To enhance the applicatory character of the outcomes, the selection of the variables was kept relevant to the conditions that actually hamper/are relevant to bioprocesses run with *Y. lipolytica*. Likewise, the target functionalities, gauging the relevance of the TFs’ programs, were kept practical. Consequently, we developed a streamlined protocol for high-throughput *Y. lipolytica* cultivation that enables reliable assessment of the phenotypes, and provided evidence for the TF-driven programs worth hijacking.

Crucially, we identified the ‘omni-booster’ of r-Prots synthesis in *Y. lipolytica* – Klf1, which has not been earlier connotated with this functionality. And found no evidence of Msn2/4s’ usefulness in enhancing *Y. lipolytica*’s stress resistance. We discovered several TFs that hold a promise of capturing the ‘holy grail’ of *Y. lipolytica*-based bioprocesses—resistance to fluctuations in OA/growth under oxygen limitation (TF036, Jmc2, TF011, and Dal81), and sustained protein synthesis under inorganic nitrogen supply (Hoy1). These findings are the starting point for the following detailed studies underway.

With the progress in high-throughput cloning, cultivation, and analytics, the processing of large-scale data and their interpretation imposes a challenge. Withdrawal of biological sense from extensive sets of numerical data is sometimes facilitated by arranging these data in queryable and filtrable databases. Completion of our experimental design covering all the *Y. lipolytica* bioprocesses-relevant variables, left us with such a problem, and prompted us to follow the proposed solution. YaliFunTome database was the answer to the arose challenge. We invite all the Yeast Society Members, especially those working with non-*S. cerevisiae* species, to browse the database in search of responses elicited by the TF homologs from *Y. lipolytica*. Our experience shows that the results coming from the non-conventional yeast may be more relevant for many species than those coming from the model.

### Supplementary Information

**Additional file 1: Figure S1.** Recombinant protein production by *Y. lipolytica* strains engineered in genes encoding selected Transcription Factors by their overexpression (OE) or deletion (KO). **Figure S2**. Microscopic images of *Y. lipolytica* strains overexpressing one of the selected Transcription Factors Azf1 (YALI0A16841g), Mhy1 (YALI0B21582g), Msn4 (YALI0C13750g), and the control strain. **Figure S3.** Factor’s contribution rankings in terms of growth, r-Prots synthesis, and normalized measure of r-Prots synthesis based on mathematical models developed using FC values readouts, for TFs: TF036 (YALI0D20460g), Jmc2 (YALI0B14443g), TF011 (YALI0B20944g), Dal81 (YALI0D02783g), and Hoy1 (YALI0A18469g). Ranking tables are color-coded according to a convention presented in Fig. 1. Percentage contribution values discussed in the manuscript are bolded.

**Additional file 2: Table S1.** List of the 125 TFs over-expressed in *Y. lipolytica* strains used in this study.

## Data Availability

The datasets generated and/or analyzed during the current study are presented directly in the manuscript, online database https://sparrow.up.poznan.pl/tsdatabase/, and as Additional materials.
