# Supplementary material for: ‘Mother(Nature) knows best’ – hijacking nature-designed transcriptional programs for enhancing stress resistance and protein production in Yarrowia lipolytica; presentation of YaliFunTome database
Source: Microb Cell Fact. 2024 Jan 18;23:26. doi: 10.1186/s12934-023-02285-x (PMC10797999; doi:10.1186/s12934-023-02285-x)
Supplement: Supplementary file 1 — Additional file 1: Figure S1. Recombinant protein production by Y. lipolytica strains engineered in genes encoding selected Transcription Factors by their overexpression (OE) or deletion (KO). Figure S2. Microscopic images of Y. lipolytica strains overexpressing one of the selected Transcription Factors Azf1 (YALI0A16841g), Mhy1 (YALI0B21582g), Msn4 (YALI0C13750g), and the control strain. Figure S3. Factor’s contribution rankings in terms of growth, r-Prots synthesis, and normalized measure of r-Prots synthesis based on mathematical models developed using FC values readouts, for TFs: TF036 (YALI0D20460g), Jmc2 (YALI0B14443g), TF011 (YALI0B20944g), Dal81 (YALI0D02783g), and Hoy1 (YALI0A18469g). Ranking tables are color-coded according to a convention presented in Fig. 1. Percentage contribution values discussed in the manuscript are bolded. [file 12934_2023_2285_MOESM1_ESM.pdf]

Supplementary Figures

Figure S1.

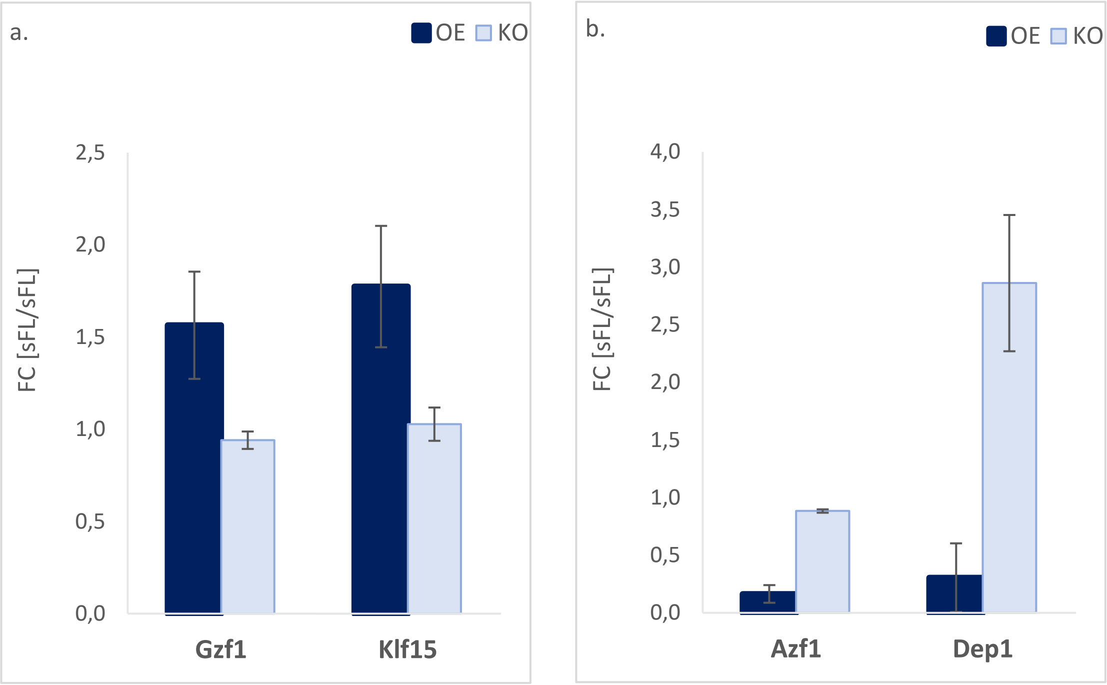

Figure S1. Recombinant protein production by Y. lipolytica strains engineered in genes encoding selected Transcription Factors by their overexpression (OE) or deletion (KO). The engineered genes were: Gzf1 (YALI0D20482g), Klf1 (YALI0D05041g), Azf1 (YALI0A16841g), Dep1 (YALI0F05896g). The OE strains were constructed as described in (Leplat et al. 2015). The KO strains were constructed using a CRISPR-Cas9 system described in (Larroude et al. 2020). The constructed strains were cultured for 48 h in the medium composed as follows [g L^{-1}]: yeast nitrogen base, 5.1 (Sigma-Aldrich); ammonium sulfate, 15, (POCH); glucose, 25 (POCH); buffered with 0.2 M maleic acid at pH 5.0. The results are presented as Fold Change values of specific Fluorescence from RedStarII [FL / OD600] of the TF-engineered strain over sFL of the control strain Y2810 (expressing RedStarII) [sFL/sFL]. sFL – fluorescence intensity normalized per biomass. FC – ratio between sFL for the TF-engineered strain over the control strain. Error bars show ± SD from three biological replicates.

Figure S2.

Azf1 Mhy1 Msn4

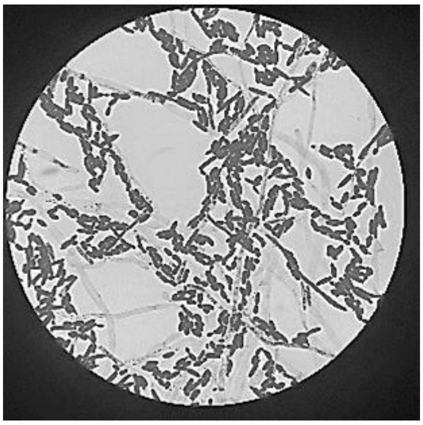

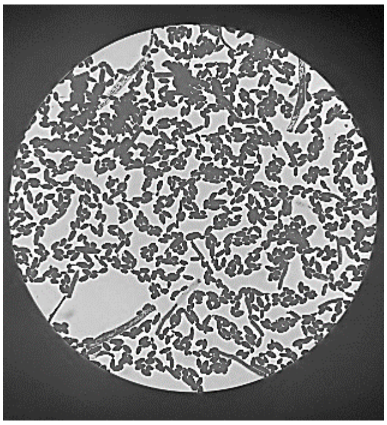

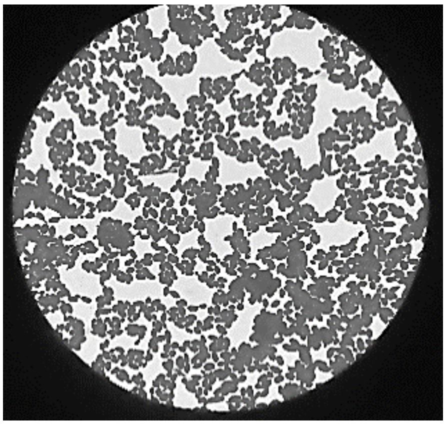

Y2810

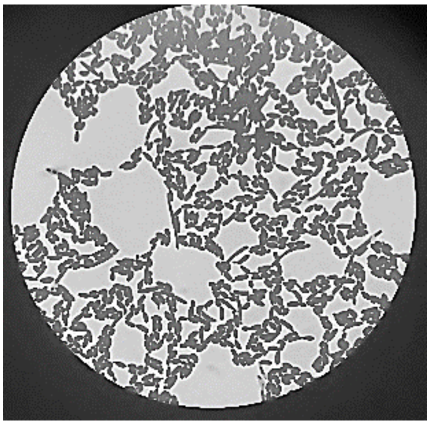

Figure S2. Microscopic images of Y. lipolytica strains overexpressing one of the selected Transcription Factors Azf1 (YALI0A16841g), Mhy1 (YALI0B21582g), Msn4 (YALI0C13750g), and the control strain. The yeasts' biomass was prepared by their culturing over 24 h in liquid YPD medium ([g L^{-1}]: yeast extract, 10 (Biomaxima); peptone, 15, (Biomaxima); glucose, 20 (POCH). Biomass was briefly centrifuged, washed in sterile saline solution, and placed on a microscope slide to prepare fixed preparation stained with crystal violet. The images were taken under 1000x magnification using a Zeiss Primo Star microscope.

Figure S3.

		growth	Total r-Prot	Normalized r-Prot
TF036 (YALI0D20460g)	pH	0,3	1,1	
	Temp	1,1		
	OA	**14,6**	12,4	
	C	3,5		1,6
	N	3,3	1,8	1,1
Jmc2 (YALI0B14443g)	pH			
	Temp		1,0	1,3
	OA	**10,6**	2,9	2,5
	C	1,3	0,5	1,0
	N	0,7		1,4
TF011 (YALI0B20944g)	pH			
	Temp		1,5	1,5
	OA	**7,3**	5,4	
	C	0,8	0,8	
	N	0,4	1,3	
Dal81 (YALI0D02783g)	pH	0,6		0,7
	Temp	3,0	2,0	
	OA	**2,7**	0,6	0,5
	C		0,7	
	N			0,4
Hoy1 (YALI0A18469g)	pH	11,1	2,1	3,7
	Temp	3,3	1,1	0,5
	OA	2,7	1,0	
	C	1,1	1,5	
	N		4,7	**7,2**

Figure S3. Factor's contribution rankings in terms of growth, r-Prots synthesis, and normalized measure of r-Prots synthesis based on mathematical models developed using FC values readouts, for TFs: TF036 (YALI0D20460g), Jmc2 (YALI0B14443g), TF011 (YALI0B20944g), Dal81 (YALI0D02783g), and Hoy1 (YALI0A18469g). Ranking tables are color-coded according to a convention presented in **Figure 1**. Percentage contribution values discussed in the manuscript are bolded.
