# Supplementary material for: ‘Mother(Nature) knows best’ – hijacking nature-designed transcriptional programs for enhancing stress resistance and protein production in Yarrowia lipolytica; presentation of YaliFunTome database
Source: Microb Cell Fact. 2024 Jan 18;23:26. doi: 10.1186/s12934-023-02285-x (PMC10797999; doi:10.1186/s12934-023-02285-x)
Supplement: Supplementary file 2 — Additional file 2: Table S1. List of the 125 TFs over-expressed in Y. lipolytica strains used in this study. [file 12934_2023_2285_MOESM2_ESM.pdf]

Supplementary Table S1

List of the 125 TFs over-expressed in *Yarrowia lipolytica*'s strains used in this study. In bold – TFs whose function has been experimentally evidenced. Assigned names and functions are provided where available; or based on blastp similarity search.

Yali number	TF number	assigned name	Putative / known function
YALI0A10637g	TF001		
YALI0A12925g	TF002		
YALI0B00660g	TF003		
YALI0B06853g	TF004	PUT3	36% identity to *S. cerevisiae* PUT3 proline utilization trans-activator
YALI0B09713g	TF005	PPR1	33% identity to *S. cerevisiae* pyrimidine pathway regulatory protein PPR1
YALI0B12716g	TF006	**HAC1**	Transcriptional activator of genes involved in Unfolded Protein Response (UPR)
YALI0B12716g	TF007	**HAC1**	Transcriptional activator of genes involved in Unfolded Protein Response (UPR)
YALI0B14443g	TF009	JMC2	JmjC domain family histone demethylase which promotes global demethylation of H_3K_4
YALI0B19602g	TF010	**MGF2**	Mycelial growth factor 2
YALI0B20944g	TF011		
YALI0C01375g	TF012		
YALI0C03564g	TF013		
YALI0C05995g	TF014		
YALI0C07821g	TF015		glucose transport transcription regulator RGT1-related
YALI0C09009g	TF016		
YALI0C09482g	TF017		
YALI0C12364g	TF018	NRG1	75% identity to *S. cerevisiae* NRG1 transcriptional repressor for glucose repression of STA1 gene expression
YALI0C13178g	TF019		
YALI0C15202g	TF020		
YALI0C16390g	TF021		
YALI0C16863g	TF022	SKO1	ATF/CREB family transcription factor, repressor that mediates HOG pathway-dependent regulation of osmotic stress response, involved in protection from oxidative damage
YALI0C22990g	TF023		56% identity to *Sugiyamaella lignohabitans* ASG1 general activator of stress genes
YALI0D01353g	TF024		
YALI0D01419g	TF025		
YALI0D04466g	TF026		
YALI0D06193g	TF027		
YALI0D06952g	TF028		
YALI0D07744g	TF029	**YAP-like**	TF involved in pH-dependent dimorphic transition
YALI0D10681g	TF031		

YALI0D13904g	TF032		44% identity to *Sugiyamaella lignohabitans* leucine-responsive transcription regulator, TF that regulates genes involved in branched-chain amino acid biosynthesis and ammonia assimilation
YALI0D14520g	TF033	**SKN7**	Transcription factor involved in the activation of osmotic and oxidative stress
YALI0D20460g	TF036	LAC9	31% identity to *Kluyveromyces lactis* LAC9 positive regulatory protein, that controls induction of the lactose-galactose regulation
YALI0D20482g	TF037	**GZF1**	Nitrogen catabolic enzyme regulator protein, INCREASE R-PROT SYNTHESIS
YALI0D23045g	TF038	AHR1	35% identity to *C. albicans* AHR1 adhesion and hyphal regulator 1
YALI0D23749g	TF039		
YALI0E03410g	TF040		
YALI0E05555g	TF041	**GZF4**	GATA-zinc finger transcription factor 4, box A-binding factor
YALI0E07942g	TF042	**MIG1**	Controls genes involved in beta-oxidation, acts as repressor of genes with carbon response elements
YALI0E10681g	TF043		
YALI0E15510g	TF044		
YALI0E17215g	TF045		
YALI0E17721g	TF046		Phosphatidylinositol N-acetylglucosaminyltransferase subunit P
YALI0E18161g	TF047		
YALI0E18656g	TF048		
YALI0E24277g	TF049		
YALI0E30789g	TF050		
YALI0E31383g	TF051		
YALI0E31669g	TF052		
YALI0E31757g	TF053	BRG1	61% identity to *C. albicans* BRG1 biofilm regulator 1
YALI0F01562g	TF054	**EUF1**	Transcription factor mediating expression of erythritol synthesis genes
YALI0F03157g	TF055	MET32	44% identity to *S. cerevisiae* MET32 auxiliary transcriptional regulator of sulfur amino acid metabolism. Involved in the transcriptional activation of MET28. Regulation of the methionine biosynthetic genes and sulfate assimilation and sulfonate metabolism
YALI0F05104g	TF057	TFIIIA	34% identity to S. *cerevisiae* PZF1 general transcription factor IIIA
YALI0F05126g	TF058		
YALI0F06072g	TF059		
YALI0F09361g	TF060	U4/U6.U5 component	57% identity to *Schizosaccharomyces pombe* U4/U6.U5 tri-snRNP, spliceosomal complex – may play a role in mRNA splicing
YALI0F13695g	TF062		
YALI0F15037g	TF063		
YALI0F16599g	TF064		

YALI0F18788g	TF067	RLM1	68% identity to *S. cerevisiae* RLM1<br>May function as a transcription factor downstream of MPK1 - is subject to activation by the MPK1 mitogen-activated protein kinase pathway. At least some RML1 target genes are involved in cell wall biosynthesis.
YALI0E13948g	TF068	**HSF1**	Heat shock transcription factor, INCREASE R-PROT SYNTHESIS
YALI0E31845g	TF069	PRZ1	49% identity to *Schizosaccharomyces pombe* PRZ1 involved in the regulation of calcium ion homeostasis. Binds to the calcineurin-dependent response element.
YALI0E11693g	TF070		
YALI0E10131g	TF071		
YALI0C22682g	TF072	**GZF3**	GATA-zinc finger transcription factor 3, TF acting as a repressor for the nitrogen catabolite repression genes
YALI0B15818g	TF073	**SterTF**	Sterol transcription factor, regulating sterol biogenesis
YALI0D09757g	TF074		AP-1-like transcription factor
YALI0F05346g	TF075		
YALI0D02475g	TF076		
YALI0B08206g	TF077	**CRF1**	Copper resistance protein transcriptional regulator
YALI0D05005g	TF078		
YALI0D12628g	TF079	**POR1**	Primary oleate regulator 1 - transcriptional activator regulating genes involved in fatty acid utilization
YALI0D04785g	TF080	SFL1	56% identity to *Candida albicans* SFL1<br>TF acting as repressor of filamentous growth and flocculation. Antagonizes functions of SFL2 and FLO8
YALI0C02387g	TF083	**YAS1**	Transcription factor essential for cytochrome p450 induction in response to alcanes, heteromeric Yas1p/Yas2p complex transcription factor
YALI0B08734g	TF084	REI1	37% identity to *S. cerevisiae* cytoplasmic pre-60S factor REI1 involved in maturation of the ribosomal 60S subunit
YALI0C11858g	TF085		
YALI0B04510g	TF086		
YALI0E01606g	TF087		
YALI0F22649g	TF088		
YALI0C06842g	TF089	MCM1	90% identity to *C. albicans* MCM1 transcription factor for morphogenesis<br>Transcription factor that is recruited by AHR1 to the promoters of genes involved in biofilm formation, which include several key adhesion genes. Plays an important role in cell adhesion, hyphal growth and virulence. Implicated in the regulation of opaque-phase-specific gene expression
YALI0A19778g	TF090	RES2/PCT1	33% identity to *S. pombe* cell division cycle-related protein Res2/Pct1

YALI0E27742g	TF091	GCN4	41% identity to *S. cerevisiae* general control transcription factor<br>Master transcriptional regulator that mediates the response to amino acid starvation
YALI0D17988g	TF093		
YALI0B15312g	TF094		30% identity to *S. cerevisiae* JJJ1 - DnaJ-like protein
YALI0B21582g	TF095	**MHY1**	Msn2/Msn4-like protein, a key regulator of yeast-to-hypha dimorphic transition but not stress response, regulates both alkaline-pH and glucose-induced filamentation
YALI0D15334g	TF096		
YALI0F03135g	TF097		
YALI0D01463g	TF098	CRZ1	64% identity to *S. cerevisiae* CRZ1 transcription regulator involved in the regulation of calcium ion homeostasis
YALI0A18469g	TF099	**HOY1**	Homeobox protein, a positive regulator of hyphae formation; deletion of Hoy1 suppresses filament formation, overexpression causes growth defects
YALI0E20449g	TF100	YOX1	53% identity to *S. cerevisiae* YOX1 homeobox protein - transcriptional repressor required to restrict transcription of ECB-dependent genes to the G1/M phase by repressing their transcription
YALI0D13068g	TF101	BUD20	59% identity to *S. cerevisiae* BUD20 bud site selection protein 20 - positioning the proximal bud pole signal; protein required for ribosome assembly: involved in pre-60S ribosomal particles maturation by promoting the nuclear export of the 60S ribosome
YALI0C18667g	TF102		Multidrug resistance regulator 1
YALI0B22176g	TF103		
YALI0E16973g	TF104		
YALI0B13354g	TF105		
YALI0F11979g	TF106		
YALI0C13750g	TF107	**MSN4**	General stress response, regulates tolerance to acid-induced stress
YALI0F25861g	TF108	RPN4	46% identity to *S. cerevisiae* RPN4 transcription factor regulating proteasomal genes
YALI0E16577g	TF109	**GZF5**	Non-genuine GATA-zinc finger transcription factor
YALI0D24167g	TF110	CBF1	40% identity to *S. cerevisiae* CBF1 centromere binding factor 1, required for chromosome stability and methionine prototrophy. It is involved in chromosomal segregation
YALI0C12639g	TF111	SWI6	32% identity to *S. cerevisiae* SWI6 part of a complex involved in cell-cycle-dependent transcription. SWI4 and SWI6 are required for formation of the cell-cycle box factor-DNA complex
YALI0C19063g	TF112		
YALI0E25960g	TF113	SWI1	28% identity to *S. cerevisiae* SWI/SNF chromatin-remodeling complex subunit SWI1
YALI0D01573g	TF115	**MGF1**	Mycelial growth factor

YALI0F21923g	TF116		
YALI0E10087g	TF117		
YALI0D02783g	TF118	DAL81	40% identity to *S. cerevisiae* (and 46% to *Komagataella phaffi*i) DAL81 transcription factor for allantoin and GABA catabolic genes<br>Positive regulator of genes in multiple nitrogen degradation pathways (GABA, urea, arginine and allantoin)
YALI0F13321g	TF119		
YALI0F17424g	TF120	HAP1	40% similarity to heme-responsive TF for oxygen sensing and signaling in *S. cerevisiae*<br>Involved in regulation of transcription in response to hypoxia
YALI0C18645g	TF121		
YALI0C19151g	TF122	CAT8	63% identity to *S. cerevisiae* CATabolite repression TF 8, inducer of gluconeogesis<br>Binding to carbon source responsive elements in the absence of glucose (contrary to Mig1)
YALI0F11487g	TF123	SFP1	77% identity to *S. cerevisiae* SFP1 nutrient- and stress-responsive activator of ribosome biogenesis genes
YALI0A16841g	TF124	**AZF1**	44% similarity to *S. cerevisiae* (and 48% to *Cyberlindnera jadinii*) AZF1 asparagine-rich zinc finger protein - TF that regulates carbon metabolism in yeast and cell wall organization – in the presence of glucose activates genes involved in growth and carbon metabolism, in nonfermentable carbon sources, activates genes involved in maintenance of cell wall integrity
YALI0B05478g	TF125	STP2	44% identity to *S. cerevisiae* STP2 transcription factor involved in the regulation of gene expression in response to extracellular amino acid levels. Synthesized as latent cytoplasmic precursor, which becomes proteolytically activated and relocates to the nucleus, where it induces the expression of subservient genes. Binding to promoters is facilitated by DAL81 (By similarity).
YALI0D05041g	TF126	KLF1	Krueppel-like factor 15,<br>regulates the expression of genes for gluconeogenic and amino acid-degrading enzymes<br>blastp search 23% similarity: Transcriptional activator of fatty acid utilization, partial [Gamsiella multidivaricata]
YALI0E18304g	TF127		
YALI0F05896g	TF128	**DEP1**	34% identity to *S. cerevisiae* DEP1 - component of the RPD3C(L) histone deacetylase complex (HDAC) responsible for the deacetylation of lysine residues on the N-terminal part of the core histones (H2A, H2B, H3 and H4). Histone deacetylation gives a tag for epigenetic repression and plays an important role

			in transcriptional regulation, cell cycle progression and developmental events.<br>Repressor of phospholipid synthesis
YALI0C14784g2	TF130	**YAS3sf**	Short form of transcriptional repressor of ALK genes, de-repressed on alkanes
YALI0B05038g	TF131	**ZNC1**	ZNC1 Zinc finger transcriptional factor regulating yeast to hyphae transition
Yali0F15543g	TF134		
Yali0E14971g	TF135		
Yali0F18326g	TF137		8-member protein family; sterol uptake control
Yali0E05577g	TF139		8-member protein family; sterol uptake control
Yali0B08360g	TF140		8-member protein family; sterol uptake control
Yali0C20977g	TF141		8-member protein family; sterol uptake control
Yali0C13794g	TF142		8-member protein family; sterol uptake control
